# Understanding Primary Care Physician Perspectives on the Diagnosis and Management of Non-Alcoholic Fatty Liver Disease: A Qualitative Study

**DOI:** 10.1177/00469580241241272

**Published:** 2024-03-26

**Authors:** Natalia I. Heredia, Jemima C. John, Sonia Singh, Jessica P. Hwang, Larkin L. Strong, Maya Balakrishnan, Lorna H. McNeill

**Affiliations:** 1 Department of Health Promotion & Behavioral Sciences, The University of Texas Health Science Center at Houston School of Public Health, Houston TX, USA; 2 Center for Health Equity, The University of Texas Health Science Center at Houston School of Public Health, Houston TX, USA; 3 Department of Epidemiology, The University of Texas Health Science Center at Houston School of Public Health, Houston TX, USA; 4 The University of Texas Health Science Center at Houston, Houston TX, USA; 5 Department of General Internal Medicine, The University of Texas MD Anderson Cancer Center, Houston, TX, USA; 6 Health Disparities Research, The University of Texas MD Anderson Cancer Center, Houston, TX, USA; 7 Section of Gastroenterology and Hepatology, Baylor College of Medicine, Houston, TX, USA

**Keywords:** qualitative research, interviews, non-alcoholic fatty liver disease, primary care physicians, family medicine, internal medicine

## Abstract

Primary care physicians (PCPs) are well suited to manage patients with non-alcoholic fatty liver disease (NAFLD), but the limited, existing research suggests inadequate knowledge about the natural history, diagnostic methods, and management of NAFLD. The purpose of this qualitative study is to further understand the knowledge and practices for the diagnosis and management of NAFLD among PCPs. We conducted in-depth interviews with PCPs in the Greater Houston area, addressing current clinical practices used for diagnosing and managing NAFLD, as well as the perceptions of the PCPs regarding the burden of NAFLD on patients. We recorded interviews, transcribed them, coded transcripts, and identified patterns and themes. The interviewed PCPs (n = 16) were from internal or family medicine, with a range of experience (1.5-30 years). We found variations in NAFLD diagnosis and management across practices and by insurance status. Patients with abnormal liver imaging who had insurance or were within a safety-net healthcare system were referred by PCPs to specialists. Uninsured patients with persistently elevated liver enzymes received lifestyle recommendations from PCPs without confirmatory imaging or specialist referral. The role of PCPs in NAFLD management varied, with some helping patients set dietary and physical activity goals while others provided only general recommendations and/or referred patients to a dietitian. The diagnosis and management of NAFLD vary widely among PCPs and may be impacted by patients’ insurance status and clinic-specific practices. The increasing burden of NAFLD in the U.S. medical system highlights the need for more PCPs involvement in managing NAFLD.

**What do we already know about this topic?**
Non-alcoholic fatty liver disease (NAFLD) is the most prevalent form of liver disease in the U.S., yet many primary care physicians (PCPs) may have a limited understanding of NAFLD diagnosis, prognosis, and management.
**How does your research contribute to the field?**
We found that among PCPs, the diagnosis and management of NAFLD varies widely and may be influenced by patients’ insurance status and clinic-specific resources and practices.
**What are your research’s implications toward theory, practice, or policy?**
Given the growing burden of NAFLD in the U.S. medical system, PCPs may need further training and institutional support to ensure equal access to diagnosis and effective management for all patients with NAFLD.

## Introduction

Non-alcoholic fatty liver disease (NAFLD) involves the accumulation of excess fat in the liver and is considered to be the hepatic complication of metabolic syndrome.^1-3^ NAFLD is a critical public health issue in the 21st century, with a prevalence that has nearly doubled in the last 3 decades.^4^ The estimated prevalence of NAFLD among U.S. adults is approximately 36%,^3^ however, this rate is even higher among Hispanic/Latino adults (42%)^5^ who have a genetic predisposition to the condition.^6^ NAFLD is closely associated with obesity and type 2 diabetes^7,8^ and is a significant factor in the increasing incidence of liver cancer and liver transplants in the United States (U.S.).^9^ Weight loss is recommended for NAFLD/non-alcoholic steatohepatitis (NASH) patients,^10^ and importantly, improved diet and physical activity have both singular and additive effects in slowing or reversing fibrosis progression even in the absence of weight loss.^11-14^

There are several factors that currently make it difficult to fully address the medical needs of patients with NAFLD. In recent years, there has been a growing trend to empower primary care physicians (PCPs) to manage the majority of NAFLD cases, reserving specialist referrals for more complex or advanced cases. However, prior studies have indicated potential gaps in PCPs’ knowledge of this condition.^15-18^ The absence of pharmacotherapy for NAFLD leaves doctors with the option of managing comorbidities such as type 2 diabetes or high cholesterol through medication (a strategy they are comfortable with) and advising weight loss (a strategy they may lack adequate training for).^19-21^ To enable PCPs to fully take on the frontline role of caring for NAFLD effectively, it is important to understand their perceptions of the condition and their perceived role in diagnosing and managing the disease. This information can help develop interventions to train PCPs on various strategies to manage NAFLD. The applicability of existing qualitative research on this topic with PCPs is limited as it was performed mainly in the UK,^22-24^ which has a markedly different healthcare system and patient population from the U.S. Therefore, we undertook this study to probe PCPs’ knowledge of NAFLD and their experience with the process of diagnosing and managing NAFLD through in-depth interviews. We conducted this study in Houston, Texas, which is situated in a state with a very high prevalence of NAFLD^25^ and with a large Hispanic/Latino population (45%),^26^ currently the group with the highest prevalence of NAFLD in the U.S.^5,27^

## Methods

We conducted a qualitative study using in-depth interviews with PCPs in Houston, Texas. Interviews were conducted in 2020 until we reached data saturation,^28^ which occurred with 16 interviews. We recruited a convenience sample of PCPs by word-of-mouth, with invitations sent via email. We sampled to recruit from various clinical settings, including the safety net system, federally qualified health centers (FQHCs), the practice arm of an academic medical center, a private clinic, and clinics with a large Hispanic/Latino patient population, due to the aforementioned burden. The Institutional Review Boards at The University of Texas MD Anderson Cancer Center and the University of Texas Health Science Center at Houston reviewed and approved the study (HSC-SPH-21-0360).

The research team developed a semi-structured, open-ended interview guide to explore physicians’ knowledge and perceptions of NAFLD diagnosis and management (Supplemental Table 1). We also included probes and prompts, as these are important to achieve saturation.^29^ The interview guide was based on a literature review performed to identify gaps in knowledge about physician perceptions. The guide was prepared in consultation with two physician-scientists with content expertise in NAFLD and/or liver cancer prevention and reviewed by two qualitative methodologists.

Interviews were conducted in-person at the clinics prior to the global COVID-19 pandemic and virtually afterward. Each session lasted approximately 30 to 45 min. First, we completed written informed consent, followed by a brief demographic questionnaire. The questionnaire included questions about sex, race and ethnicity, medical specialty, and years of experience practicing medicine. All interviews were audio recorded with participant consent and transcribed verbatim by a professional transcription service. Participants received a $25 gift card as compensation for their time.

### Analyses

We used Nvivo 12 Pro (QSR International, Burlington, Massachusetts) to code and analyze the interviews using thematic analysis.^30^ We developed an initial set of structural codes meant to capture the topics addressed by the semi-structured interview guide. Then, the team read the transcripts and developed a tentative set of inductive codes. First, five transcripts were coded using the initial codebook, and then the codes and definitions were further refined before the two coders proceeded. The modified codebook was then used to revise the previously coded transcripts and to code the remaining transcripts, with additional modifications to the codebook and the coding technique taking place at regular intervals until all transcripts were coded with the finalized coding scheme.

## Results

We completed a total of 16 interviews with PCPs, with seven from family practice and nine from internal medicine, from seven different institutions and 12 distinct clinic locations, across various types of practices (See Table 1). PCPs’ years of clinical experience varied significantly, ranging from 1.5 to 30 years (mean 15 years, median 11.5 years).

**Table 1.** Participants Demographic Characteristics (N = 16).

Variable	N (%)
Sex	
Male	5 (31)
Female	11 (69)
Specialty	
Internal medicine	9 (56)
Family medicine	7 (44)
Race/Ethnicity	
Hispanic/Latino	4 (25)
Non-Hispanic White	7 (44)
Non-Hispanic Asian	5 (31)
Practice type	
Federally qualified health center	7 (44)
Safety net system	3 (19)
Academic medical center-affiliated clinic	5 (31)
Private clinic	1 (6)

We identified the following themes, which are discussed in detail below: factors influencing NAFLD development, diagnosis of NAFLD, monitoring of NAFLD, communicating with patients about NAFLD progression, weight loss and behavioral lifestyle change recommendations, perception of patient behavior change following diagnosis, and frustration with the state of the science.

### Factors Influencing NAFLD Development

Most PCPs were not aware of the potential link between genetics and NAFLD, with one stating that “. . .I don’t know of a direct genetic link outside of just if you have the same genetic links for obesity and metabolic syndrome” (Female, 007). Nearly half of the PCPs reported observing higher rates of NAFLD in the Hispanic/Latino population compared to other racial/ethnic groups, with one PCP noting that NAFLD also tended to be more severe in this population.

Most PCPs identified factors such as inactivity, a sedentary lifestyle, and poor dietary habits, including the consumption of high-calorie and processed foods, as the primary drivers behind the initial onset of NAFLD. Several PCPs discussed the impact of social determinants of health and socioeconomic status on dietary habits and physical activity behavior. One PCP stated that “I really think that we have to look at economic status as major risk factor because tortilla and breads are a lot cheaper than fresh vegetables” (Female, 008). Another physician noted that “the environment plays an important part, such as the existence of food deserts and unsafe outdoor environments so that people cannot do physical activity” (Male, 001).

Several PCPs indicated that obesity is often an underlying factor, and one PCP stated that “especially in the obese population, when we have BMI more than twenty-five, thirty, overweight or obese patients. . .we see the extra fat that tends to build up in the liver due to causes other than alcohol use” (Female, 012). These PCPs identified chronic disease-related comorbidities, including insulin resistance, diabetes, and hypertension, as high-risk factors for NAFLD, with diabetes being specifically mentioned by most of them.

### Diagnosis of NAFLD

While PCPs did not specifically screen for NAFLD, the diagnosis was often made during annual routine check-ups that involve obtaining a comprehensive metabolic panel, which includes liver enzyme values. Therefore, these checkups play a key role in the diagnosis of NAFLD. One PCP stated that “no one’s saying ‘I’m going to do this test to screen for non-alcoholic fatty liver disease’, but because almost everybody at risk for it gets a comprehensive metabolic panel. . .they are getting screened anyway” (Male, 004). In addition to routine bloodwork, PCPs also mentioned that sensitivity or pain in the upper right quadrant of the abdomen raised their suspicion of NAFLD, prompting them to order an ultrasound for further evaluation. Lastly, the presence of comorbidities, specifically obesity and diabetes, often led most PCPs to suspect NAFLD. One PCP said that “somebody who has diabetes, somebody who has increased waist to hip ratio . . . anywhere above the normal BMI range, you suspect [NAFLD]” (Female, 007). Some PCPs expressed the importance of early identification of NAFLD because it is potentially reversible, and early detection can prevent long-term health consequences of NAFLD. In the case of elevated liver enzymes, which indicate abnormal liver function, PCPs “[ruled] out other common causes of elevated liver enzymes” (Male, 004) before ordering imaging and formally diagnosing NAFLD. Most PCPs waited for a second or third panel showing elevated liver enzymes before taking further action. PCPs mentioned that they never ordered liver biopsies and preferred to defer that decision to specialists.

#### Differences in Management Approach by Patient Resources and/or Insurance Status

Depending on the results of repeated tests of elevated liver enzymes and/or imaging, PCPs either referred the patient to a gastroenterologist or a hepatologist, or continued to manage the patient themselves. The latter approach consisted of monitoring liver enzyme levels and providing behavioral lifestyle change counseling. Patients’ insurance status and PCPs’ type of practice largely dictated this decision making. PCPs whose practices included privately insured patients or those within the safety-net healthcare system preferred referrals to liver specialists to monitor and manage NAFLD. Although safety-net health system-based PCPs had low-resource patient populations, they had access to fatty liver specialists within the system, though high demand dictates that patients can only be referred if liver tests were 150% elevated. Most PCPs practicing at FQHCs reported that they tried to avoid referring patients to specialists as much as possible due to the financial constraints of their patients, making efforts to limit referrals to only when the disease progressed beyond their ability to treat or if patients became symptomatic (indicating severe disease). In certain cases, they referred patients to a specialist once or twice but then assumed responsibility for the long-term management of the patients and ensured they followed the specialist’s recommendations.

Some PCPs in FQHC settings with uninsured patients and limited resources reported that they often discussed the issue of fat accumulation in the liver and how to manage it even without imaging confirmation or specialist input because many patients may not have access due to financial limitations. They tell patients: “‘Okay, look, you’ve got fat in your liver. You have elevated liver function tests. You need to receive treatment.’ So, we’re diagnosing it without officially diagnosing it, if that makes sense” (Male, 013). Similarly, another PCP stated that “if a patient doesn’t have as much money or depending on their level of insurance, we’re not going to order tests that I’m not going to act on or do anything differently” (Female, 005).

### Monitoring of NAFLD

The PCPs emphasized the importance of monitoring liver function following a diagnosis to prevent the progression of NAFLD to more severe outcomes such as cirrhosis or cancer. Almost all PCPs monitor the level of liver enzymes at least once a year, but the frequency of visits depends on factors such as the patient’s insurance status, NAFLD severity, comorbidities, and/or sufficient monitoring by and coordination with a liver specialist. Patients with existing comorbidities visit clinics more often, allowing PCPs to monitor NAFLD without NAFLD being the focus of the visit. One PCP said “. . .if they just had NAFLD, then I probably would not see them with the same frequency that I would see them if they had diabetes and hypertension” (Female, 006).

While a few PCPs monitored the disease progression over time using ultrasound, the majority of them did not think that another ultrasound after the initial diagnosis would be useful. One PCP said that “the ultrasound is not sensitive enough to notice changes between visits” (Male, 003). Lack of insurance and limited resources also influence clinical decision making. One participant said “. . .ultrasounds, CT scans oftentimes are too expensive for our patients to afford. . .we might usually continue to track. . .the liver enzymes and make sure that the hepatitis panel is normal. . .” (Female, 015).

### Communicating With Patients About NAFLD Progression

There were also some differences among PCPs’ approach to explaining the progression of NAFLD to their patients, especially concerning the most severe consequences (e.g., cirrhosis and liver cancer). While some PCPs thoroughly explained how NAFLD could worsen and the potential severity of the disease, others advised patients with NAFLD to take care of their health to avoid any future liver and comorbidity complications but did not explain the disease progression in detail. One PCP expressed that NAFLD “can progress to end-stage liver disease . . . I don’t know if I tell everybody that” (Female, 005). The approach to explaining the progression of liver disease was also dependent on the PCPs’ clinical experience and the duration of their practice in this field. Some PCPs were not well-versed in the progression of NAFLD because they did not see many patients with severe liver disease, and others thought that there was a lack of scientific consensus on who progresses to severe liver disease.

### Weight Loss and Behavioral Lifestyle Change Recommendations

All PCPs encouraged patients to lose weight to manage their condition because, as one participant exemplified, “the last time I looked, weight loss was still the best thing for [NAFLD]” (Male, 002). Because these comorbidities are intertwined, PCPs had similar conversations with patients who have NAFLD or only comorbidities. For example, one PCP said, “If we manage fatty liver, other comorbidities will improve. . . these are all interlinked” (Female, 012). Several PCPs recommended patients to lose 5% to 10% of their weight: “. . .we start with 5% because that’s easier for people to understand . . . we have good data to say that 5% is a meaningful number” (Female, 005). One PCP contradicted this blanket recommendation: “I don’t actually know if there’s a specific percentage I should be telling [patients] to lose . . . even if there was, I wouldn’t tell them because I don’t think that it’s very patient-centered” (Female, 014). Another PCP specified that they do not set specific weight loss targets for their patients because doing so could overwhelm them; rather, they help patients set their own achievable goals.

A handful of PCPs used motivational interviewing techniques, helping patients identify culturally adaptable and sustainable changes in their daily lives. A few PCPs helped patients set specific goals for themselves because “‘You just have to lose weight’ is not a very helpful statement – or ‘Just eat healthily’, well, what does that look like? So, I try to guide them through that” (Female, 006). In addition to healthy eating, these PCPs recommended an increase in physical activity but also asked questions about the patients’ daily activities to customize advice. Some PCPs practicing within FQHCs indicated that they help patients set these goals, add these goals to their medical records, and follow up with patients on their goals during each visit. “So you’ll give them one challenge to make or a change to make in their diet and then follow up in two months or so – one to three months – and see if they’ve been able to make that change” (Female, 009). After observing even one small change, PCPs encouraged patients to gradually make their goals more ambitious. For example, one PCP recommended “increasing the intensity of exercise. Not necessarily the time of exercise because a lot of them don’t have a lot of time outside of work free. . .gradually increasing intensity of exercise really has helped” (Female, 015). One PCP stressed the need for more extensive and widely available “education on the importance of a healthy diet” and seemed to understand the complexity behind behavior change, indicating that simply instructing someone how to eat is insufficient; instead, they need a comprehensive program that focuses on building skills and overcoming barriers: “showing, having the capacity to show them, and addressing the socioeconomic factors that prevent access to healthy food” (Female, 007). Similarly, a few PCPs emphasized that lifestyle modifications would be easier if patients had fewer barriers and greater access to resources: “I would love them to have more places to exercise” (Female, 005).

In contrast, some PCPs gave more general recommendations on lifestyle behaviors, or recommendations solely based on national guidelines, rather than tailoring to the individual. For example, one PCP “. . .encourage[d] patients to do 30 minutes of exercise five times a week at least, if not more” (Male, 003). This is because these PCPs in particular did not have lifestyle behavior-related training and preferred to refer patients to dieticians or health educators. Some also noted their lack of comfort with lifestyle recommendations: “. . .any conversation that involves predominantly diet, most of the time you don’t spend a lot of time on it because you don’t have a sense of control over it as a physician, whereas if you can give a medication, it’s nice, ‘Oh, start this medication and your disease process is going to get better’” (Female, 007). However, a patient’s lack of insurance or resources interfered with successful referrals. There was an exception to this limitation for two FQHCs and clinics within the safety-net system that provided these services in-house, free of charge.

Many PCPs advised their patients to limit or completely avoid alcohol consumption following NAFLD diagnosis in order to prevent further damage to the liver. Some PCPs may also suggest reducing alcohol consumption for weight loss purposes: “Reduction in alcohol is another good way to lose weight . . . if you stop that, you’ll lose weight” (Male, 004).

### Perception of Patient Behavior Change Following Diagnosis

The PCPs reported a variety of observations regarding the feelings and behaviors exhibited by patients after receiving a NAFLD diagnosis. Some patients were anxious and frustrated because they “. . .are surprised that their diet can cause this, and two, they kind of feel unhealthy. They feel like now they are a sick person. . .” (Female, 007). Some patients did not change their diet, physical activity, or alcohol consumption behaviors, while others exhibited a concerted effort to lose weight. Following the diagnosis, some patients became “more aware of their disease” and more “careful about their diet,” with the most common behavior changes being a reduction in carbohydrate and sugary beverage intake and the initiation of a regular walking. The concerns of patients about the diagnosis and potential long-term negative health outcomes were “a motivator to make changes in their lifestyle” (Female, 009). However, an increased perception of disease severity among patients did not always induce motivation: “Some of them do get worried and those who get worried and probably are getting concerned, they do act on it. Some of them get worried and don’t act on it. Some get worried and will go the other direction” (Female, 011). Regardless, motivation, in general, plays a crucial role for those who were successful in developing long-term healthy habits and losing weight. Several PCPs stated that patients “may have issues with motivation as well. . .there may be factors that may be preventing them from sticking to some kind of diet plan or weight-loss regimen” (Female, 016).

### Frustration with the State of the Science

Most PCPs expressed frustration with the lack of clarity regarding the standards for screening, diagnosing, or managing NAFLD. Some mentioned the lack of well-defined guidelines and limited research on NAFLD. Some PCPs conveyed their frustration and uncertainty in identifying which patients with NAFLD are more susceptible to developing progressive liver disease. One PCP mentioned that they “know the information’s out there as far as how many people have it and then progress to NASH and then cirrhosis, but more robust information on that would be nice too” (Female, 009). The PCPs were also frustrated with limited treatment options; one PCP expressed lack of confidence in prescribing the available medications for treating NAFLD, whereas another PCP explained that there is limited data on the effectiveness of existing medications, often for co-morbid conditions, for treating the disease.

## Discussion

The results of this qualitative study, which gathered the viewpoints of PCPs from family practice or internal medicine departments at various institutions in Houston, TX, enhanced our understanding of PCP knowledge of the causes and pathophysiology of NAFLD, as well as their varying approaches to diagnosis and management of the condition. Most PCPs diagnose NAFLD through bloodwork conducted as part of routine check-ups. The approaches to confirming the diagnosis and managing NAFLD vary according to patients’ financial resources and insurance coverage. Our results suggest that most FQHC-based PCPs are hesitant to refer patients with NAFLD to specialists due to concern for the financial and time constraints and potential mental burden such referrals may have on a patient. Meanwhile, safety-net system-based PCPs—despite the availability of in-house specialists at potentially reduced or no additional cost to patients—must limit referrals to severe cases of NAFLD due to high demand. This is problematic as it may be contributing to the disparities in hospitalizations and NAFLD-related mortalities seen in individuals with non-private health insurance or without insurance.^31^

All PCPs in this study said that they encouraged weight loss to manage NAFLD, as it remains the only treatment option at the present time. Many of the PCPs lacked training in lifestyle-related issues and experienced discomfort in giving nutrition advice. We also found that while some PCPs used motivational interviewing techniques such as asking open-ended questions, letting patients identify achievable goals, and following up on those goals with NAFLD patients, this was not widespread. Prior research has shown that physicians can play a powerful role in motivating and guiding patients in making behavioral change.^32,33^ Thus, trainings and/or interventions specifically for PCPs to build knowledge and confidence for delivering lifestyle behavior change recommendations are warranted. Motivational interviewing, a technique that providers can use to help patients make changes in both their physical activity and dietary behaviors,^34,35^ has been shown to improve weight loss in patients who are overweight or obese.^36,37^ While physicians may face time constraints during a clinical visit, they can still receive training on communication skills to build relationships with their patients and encourage lifestyle behavior changes.^38^

While the PCPs generally had a good understanding of how to diagnose and monitor NAFLD, some expressed frustration with the current state of the science on diagnosis and treatment. Note that this field has rapidly evolved since these interviews were conducted in 2020. For example, according to the U.S. guidelines for screening patients with comorbid conditions,^39-41^ individuals with obesity, type 2 diabetes, and/or two or more features of the metabolic syndrome should be screened for advanced fibrosis. Moreover, these guidelines provide clear instructions on which patients should be referred to specialists,^39-41^ a guideline which was previously lacking. Lastly, while lifestyle behavior changes to achieve clinically significant weight loss remains the mainstay for treatment of all patients with NAFLD, new obesity drugs have been approved with potential benefit for severe NAFLD fibrosis.^42-44^ In addition, medications for severe NAFLD fibrosis are under consideration by the FDA.^40^ Despite these improvements in guidance, PCPs remain central to managing the NAFLD epidemic. In fact, their role has only become more important in recognizing, diagnosing, triaging, and educating patients with NAFLD as frontline providers. Concerted efforts to disseminate updated guidelines and advances in NAFLD science among PCPs are vital.

## Strengths and Limitations

There are several limitations to this study that must be acknowledged. Foremost, this is a qualitative study conducted with a selected group of PCPs in Houston, TX, and therefore, we are unable to generalize our findings to a larger PCP population in the U.S. However, we conducted interviews with a diverse group of PCPs, including both men and women from different racial and ethnic groups. They also had varying years of clinical experience and worked across a range of medical institutions. Qualitative research is designed using small sample sizes. However, we ensured that we achieved saturation in our qualitative research, where similar concepts were repeated across interviews and no new concepts emerged during the final interviews. Lastly, our findings are based on self-reported survey-based assessments, and thus, may be subject to social desirability bias. Nevertheless, this is one of the few studies on the perceptions of PCPs regarding the diagnosis and management of NAFLD in the U.S. The findings can serve as a valuable foundation for future quantitative investigations and can inform the development of training programs and other interventions aimed at enhancing PCPs’ knowledge and skills in this area, including CME content, a short program on the basics of motivational interviewing and/or lifestyle behavior change, among other potential interventions, including low-cost dietary interventions for patients in low-resource clinics.

## Conclusions

The diagnosis and management of NAFLD vary widely among PCPs and may be influenced by patients’ insurance status and clinic-specific resources and practices. Given the growing burden of NAFLD in the U.S. medical system, PCPs may need further training and institutional support to ensure equal access to diagnosis and effective management for all patients with NAFLD.

## Supplemental Material

sj-docx-1-inq-10.1177_00469580241241272 – Supplemental material for Understanding Primary Care Physician Perspectives on the Diagnosis and Management of Non-Alcoholic Fatty Liver Disease: A Qualitative Study Supplemental material, sj-docx-1-inq-10.1177_00469580241241272 for Understanding Primary Care Physician Perspectives on the Diagnosis and Management of Non-Alcoholic Fatty Liver Disease: A Qualitative Study by Natalia I. Heredia, Jemima C. John, Sonia Singh, Jessica P. Hwang, Larkin L. Strong, Maya Balakrishnan and Lorna H. McNeill in INQUIRY: The Journal of Health Care Organization, Provision, and Financing
