# Supplementary material for: Understanding Primary Care Physician Perspectives on the Diagnosis and Management of Non-Alcoholic Fatty Liver Disease: A Qualitative Study
Source: Inquiry. 2024 Mar 26;61:00469580241241272. doi: 10.1177/00469580241241272 (PMC10967000; doi:10.1177/00469580241241272)
Supplement: sj-docx-1-inq-10.1177_00469580241241272 – Supplemental material for Understanding Primary Care Physician Perspectives on the Diagnosis and Management of Non-Alcoholic Fatty Liver Disease: A Qualitative Study [file sj-docx-1-inq-10.1177_00469580241241272.docx]

Supplementary Table 1. Semi-structured Interview Guide

| 1. Can you tell me in your own words about non-alcoholic fatty liver disease (NAFLD)? Probe: How is it diagnosed? What tests are generally used? What is Non-alcoholic steatohepatitis (NASH)? |
| --- |
| 1. What would make you suspect that a patient may have NAFLD? |
| 1. Do you routinely test for NAFLD in your practice? Why/Why not? |
| How do you typically diagnose/test for NAFLD in your clinic/practice? Probe: ultrasound, fibrosis biomarkers, etc. |
| 1. Tell me the reasons you think it’s important to test for NAFLD. Note: Try to elicit if he/she knows that NAFLD can progress to NASH, fibrosis, cirrhosis, HCC; that early fibrosis is reversible, while late stage is not. |
| 1. Tell me about how you explain a diagnosis of NAFLD to your patients. |
| 1. What do you typically tell your patients about the progression of NAFLD? |
| 1. What handouts or information brochures, if any, do you or your clinic offer patients on NAFLD? |
| 1. What factors do you think play a primary role in the development of NAFLD in your patients? Note: Try to understand what they attribute to the development of liver disease. Probe: Genetics, Environmental toxins or hazards, Diet, Exercise, alcohol or tobacco, Illicit drug use, Other medicines, Hepatitis virus, Race/ethnicity, culture, etc. |
| 1. What factors do you think are important to prevent the progression of NAFLD to NASH, fibrosis, cirrhosis and/or hepatocellular carcinoma? Probe: Genetics, Environmental toxins or hazards, Diet, Exercise, alcohol or tobacco, Illicit drug use, Other medicines, Hepatitis virus, Race/ethnicity, culture, etc. |
| 1. In your practice, do you currently manage NAFLD and/or monitor its progression for those who have been diagnosed? Why/Why not?    1. [*If yes*] How do you managed and/or monitor the progression of NAFLD in your patients?    2. [*If no*] Why not? Probe: need formal recommendations, need resources, etc. |
| 1. Since NAFLD often co-occurs with other conditions, such as metabolic syndrome or type 2 diabetes, how do you manage NAFLD alongside these other co-morbidities? |
| 1. What do you say to your patients about how they can manage their NAFLD? Probe: recommend diet, exercise, weight loss; refer to lifestyle modification therapists/weight loss specialists/dieticians, etc.; recommend more frequent visits to your clinic/a specialty clinic?    1. What specific recommendations do you give? |
| 1. What have you observed about the ways that NAFLD impacts your patients’ lives?    1. Has it made their life more difficult?    2. Has it made them more worried/concerned? |
| 1. Tell me about any changes your patients have made in their health behaviors after they’ve been diagnosed with NAFLD?    1. What has worked, what has not worked?    2. What do you need to help you help your patients? |
| 1. Tell me the reasons you think it’s important to monitor NAFLD in your patients. |
| 1. Do you monitor the progression of NAFLD in your patients? How so? With what frequency? |
| 1. Some ethnicities have higher rates of NAFLD than others. Tell me about your experience with the diagnoses, management and/or monitoring of NAFLD in different ethnicities. Probe: Has your experience with diagnosing and/or managing NAFLD been different with Hispanic patients than it has with other patients? |
| 1. Is there anything else you would like to share? |
